# Supplementary material for: Density fluctuations, homeostasis, and reproduction effects in bacteria
Source: Commun Biol. 2022 Apr 28;5:397. doi: 10.1038/s42003-022-03348-2 (PMC9050864; doi:10.1038/s42003-022-03348-2)
Supplement: Supplementary file 2 — Supplementary Information [file 42003_2022_3348_MOESM2_ESM.pdf]

Supplementary Information for:

Density Fluctuations, Homeostasis, and Reproduction Effects in Bacteria

S. Nemati[1], A. Singh[2], S. D. Dhuey[3], A. McDonald[4], D. Weinreich[5], A. E. Vasdekis[1]

[1] Department of Physics, University of Idaho, Moscow, ID, USA.

[2] Electrical and Computer Engineering, University of Delaware, Newark, DE, USA.

[3] Molecular Foundry, Lawrence Berkeley National Laboratory, Berkeley, CA, USA.

[4] Department of Forest, Rangeland and Fire Sciences, University of Idaho, Moscow, ID, USA.

[5] Department of Ecology and Evolutionary Biology, Brown University, Providence, RI, USA.

Supplementary Figures

Supplementary Figure 1

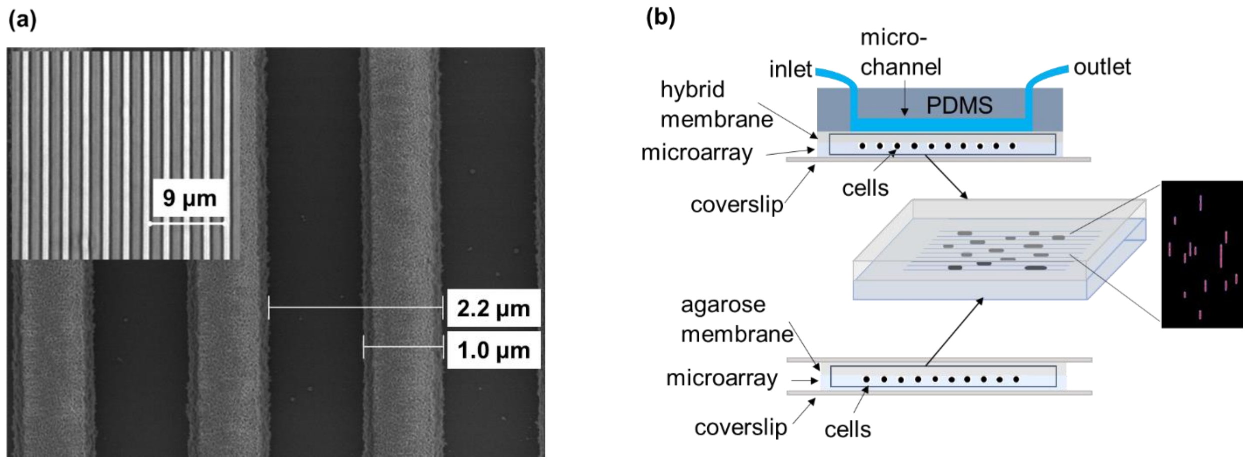

(a) Scanning Electron Microscopy (SEM) of a region of the 1D microarray in SU8 on a Si wafer (~1.2 µm linewidth where cells reside in the 1 µm spacing); *inset* displays a bright field image of the same microarrays transferred onto the BIO133 polymer. **(b)** Schematic representation of the 1D growth microarrays; *top*: cells positioned in the 1D microarrays and vertically confined via a hybrid membrane and a microfluidic system that delivers medium to the cells; *bottom*: cells positioned in a 1D microarray and confined vertically by a top-integrated agarose membrane that is doped with nutrients; *middle*: 3D representation of the cell-growth region. In all cases, imaging was performed through the bottom coverslip.

Supplementary Figure 2

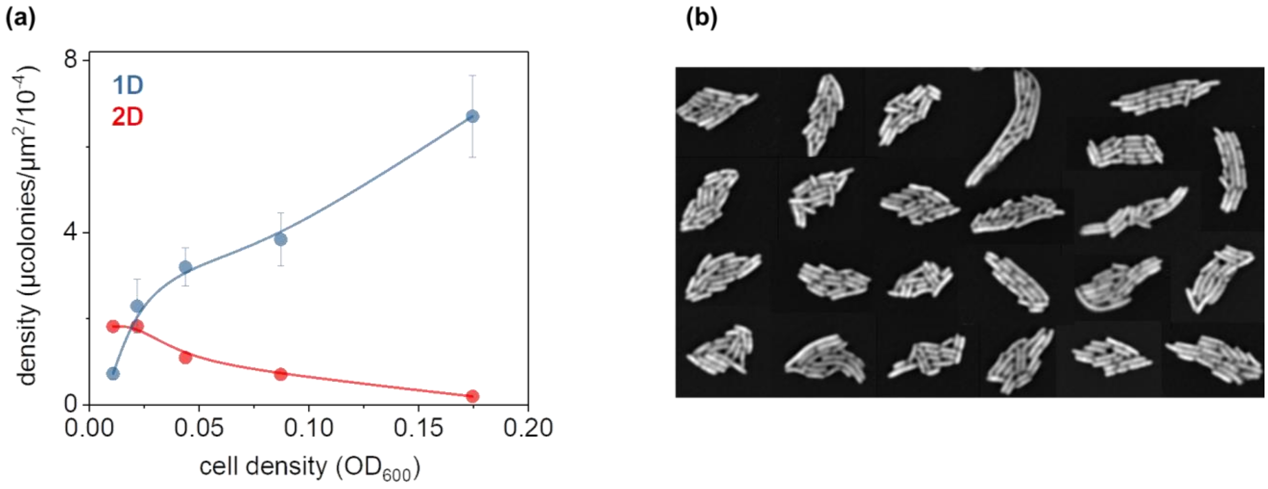

(a) Density of observations (i.e., number of observable microcolonies per unit area) of the 1D (1 μm spacing) and 2D growth assays as a function of the optical density of the deposited samples; data points represent the average and error bars the standard deviation of 3 replicates for growth of up to 16 cells; the density of observations is significantly higher for ODs equal or greater than 0.04 (two-sample t-test under the Welch Correction for ODs 0.044 ($p = 0.009$, $t = 4.78$, DF = 4), 0.088 ($p = 0.007$, $t = 5.03$, DF = 4), and 0.175 ($p = 0.002$, $t = 6.84$, DF = 4). **(b)** A representative montage of several 2D *E. coli* microcolonies illustrating shape asymmetry and underlying stochasticity.

Supplementary Figure 3

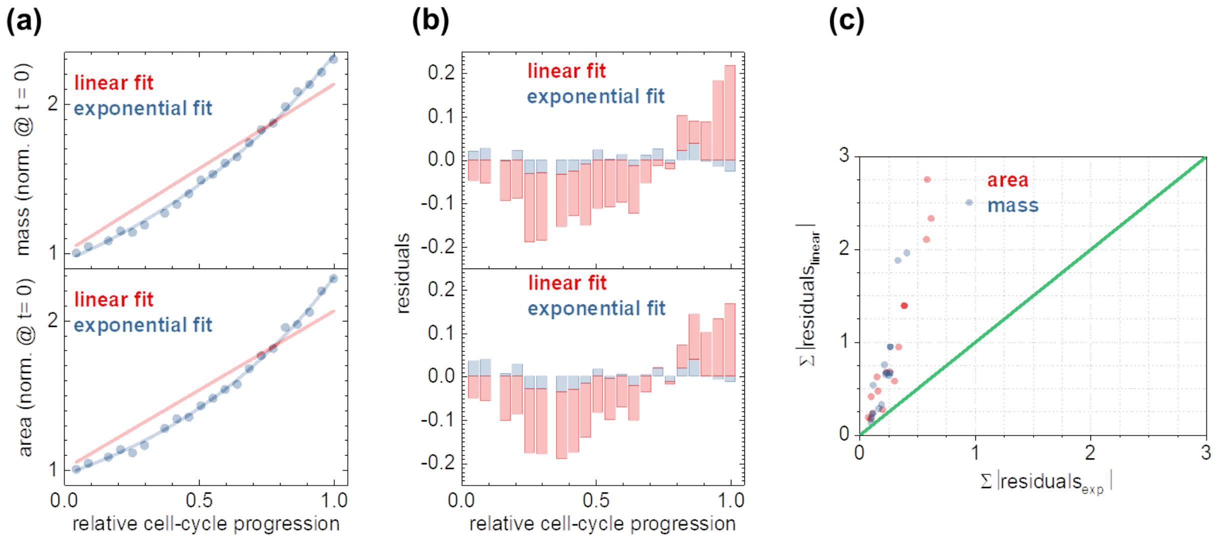

(a) Typical examples of the exponential nature of size and mass accumulation; data points represent experimental observations while the solid lines represent the linear (red) and exponential (blue) fits. **(b)** Plot of regular residuals for the curves presented in (a). **(c)** Residual sum comparison for all area and dry-mass fits presented in **Fig. 1d**; here, we specifically plot the sum of the absolute residuals for mass and area accumulation during the cell cycle (i.e., $\sum_{\text{birth}}^{\text{division}} |\text{residual}|$).

Supplementary Figure 4

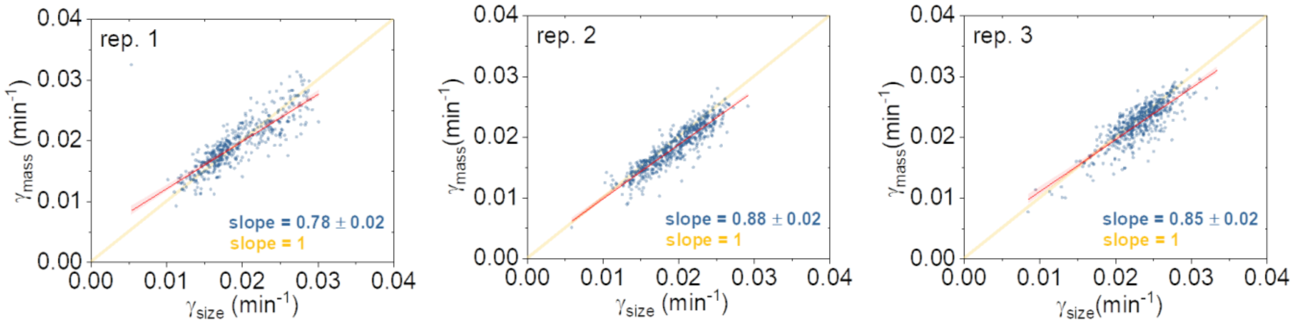

Growth differentiation with some cells optimizing size accumulation rates (γ_{size}) and some biomass accumulation rates (γ_{mass}); each graph corresponds one experimental replicate performed on different days; red line represents the linear fit (shaded areas are the 95% confidence intervals); yellow line represents a hypothetical slope of 1.

Supplementary Figure 5

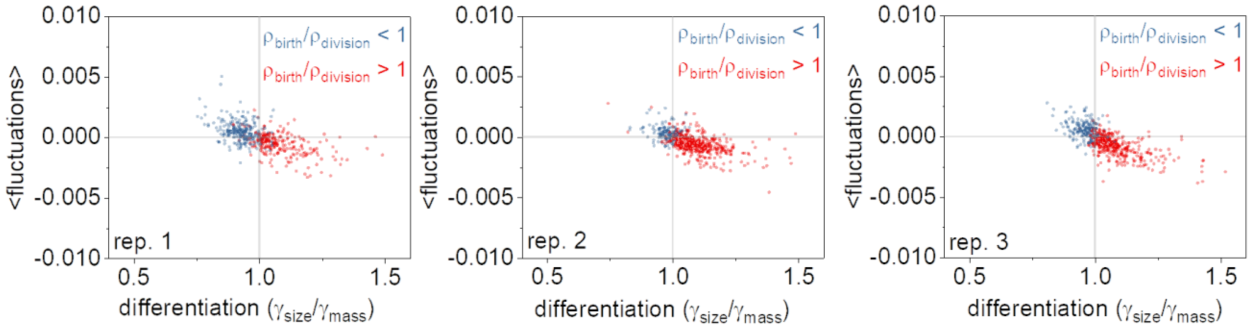

Median density fluctuations (<dρ/dt>) during growth as a function of growth differentiation ($\gamma_{size}/\gamma_{mass}$) for three replicates; corresponds to overall increasing (blue) or decreasing (red) cellular dry-density during growth.

Supplementary Figure 6

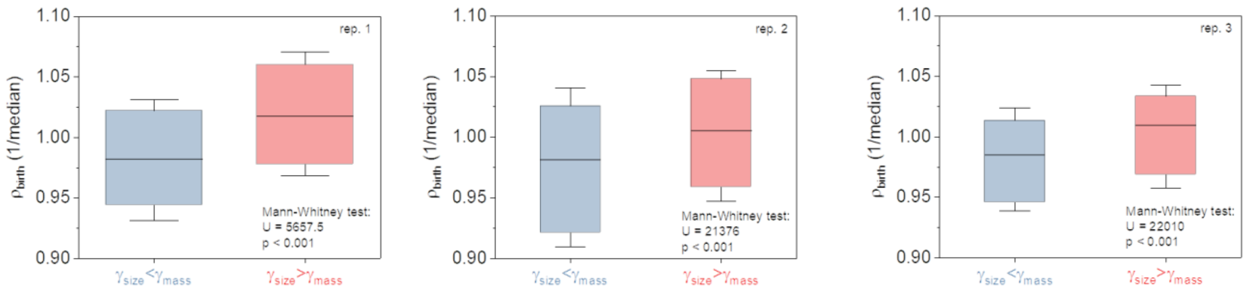

Cells born with lower density than the population median maximize biomass accumulation rates (differentiation or $\gamma_A/\gamma_M < 1$, blue), while cells born with higher than the population median density maximize area accumulation rate (differentiation or $\gamma_A/\gamma_M > 1$, red). Boxcharts represent the 25%-75% of the cumulative response of three replicates; legends summarize the result of the Mann-Whitney test, with additional tests presented in **Supplementary Table 1**).

Supplementary Figure 7

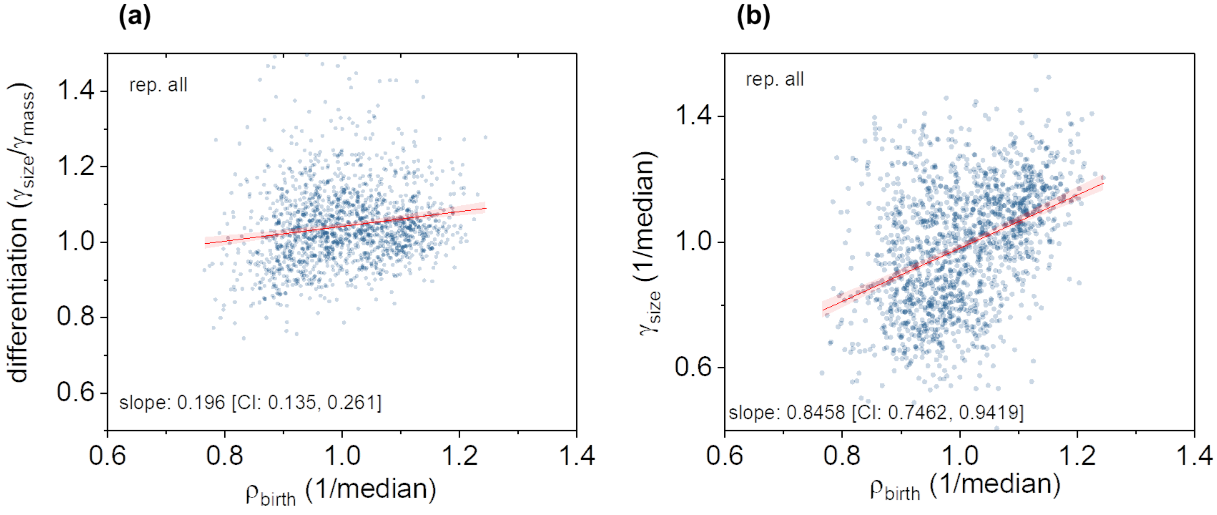

(a) Growth differentiation ($\gamma_{size}/\gamma_{mass}$) as a function of cell density at birth (ρ_i). **(b)** Growth rate by size (γ_{size}) as a function of cell density at birth (ρ_i). In both graphs, all three replicates are combined with the red line representing a linear fit, while legend denotes the linear slope and the corresponding 95% confidence intervals (CI) determined by bootstrapping.

Supplementary Figure 8

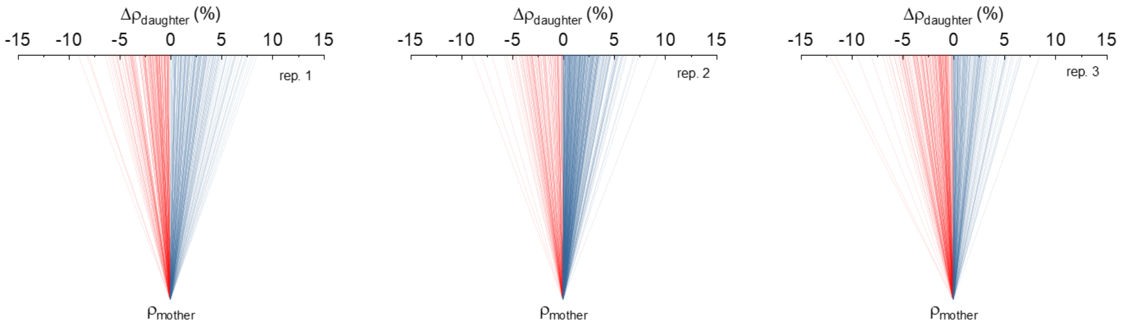

Dry-density asymmetry upon division, noted by the density differences ($\Delta\rho_{daughter}$ %) between each daughter ($\rho_{daughter\text{-}I}$ at birth) to its mother (ρ_{mother} at division). Blue (red) traces correspond to density increases (decreases) upon division and asterisks denote statistical significance (One Sample Wilcolxon Signed Rank Test, **Supplementary Table 2**) of nonzero daughter density differences from their mother. The graphs represent the response of each biological triplicate separately.

Supplementary Figure 9

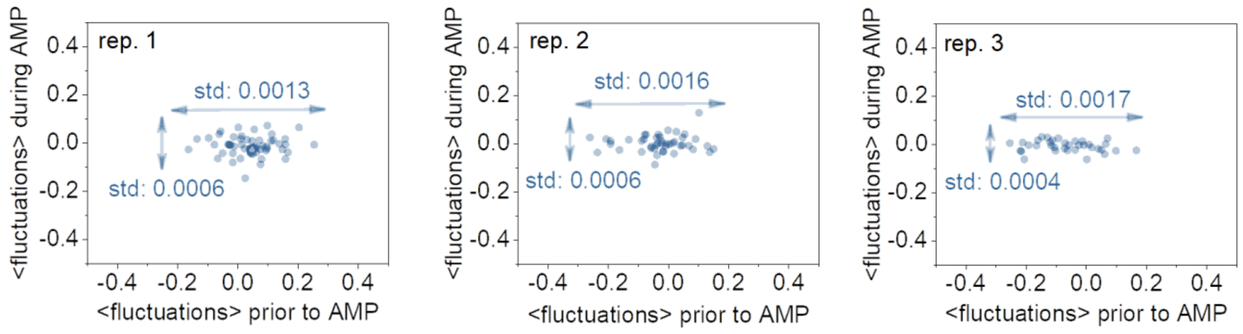

Density fluctuations disappears under the ampicillin treatment; graphs represent three separate experimental replicates; *legends* note the standard deviation of fluctuations before (*horizontal arrow*) and during the ampicillin treatment (*vertical arrow*);

Supplementary Figure 10

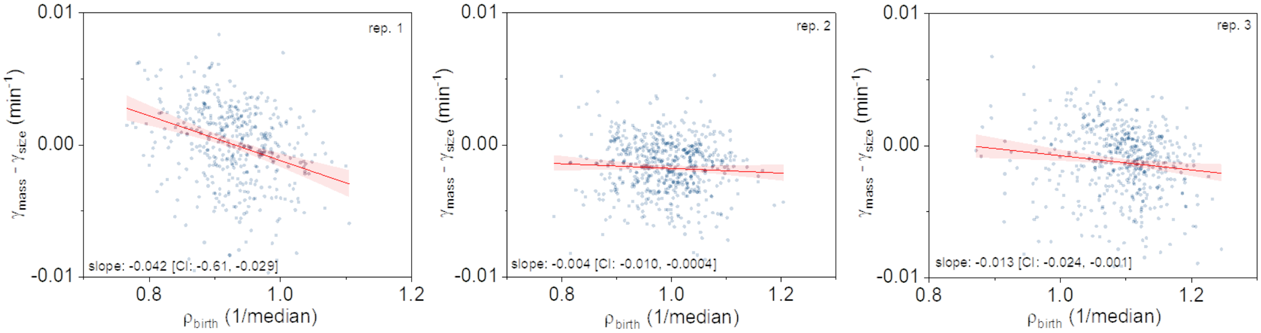

Density homeostasis during the cell cycle, evidenced by the monotonic decrease of $\gamma_{mass} - \gamma_{size}$ with respect to the newborn cell density (ρ_{birth}); each graph plots the response of each experimental replicate separately.

Supplementary Figure 11

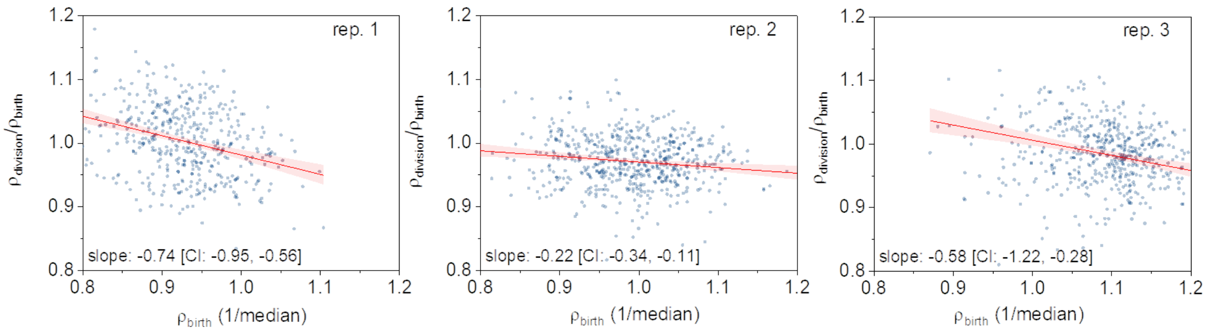

Density homeostasis during the cell cycle, with the final cell density ($\rho_{division}$) being dependent on the cellular dry-density at birth (ρ_{birth}); each graph plots the response of each experimental replicate separately.

Supplementary Figure 12

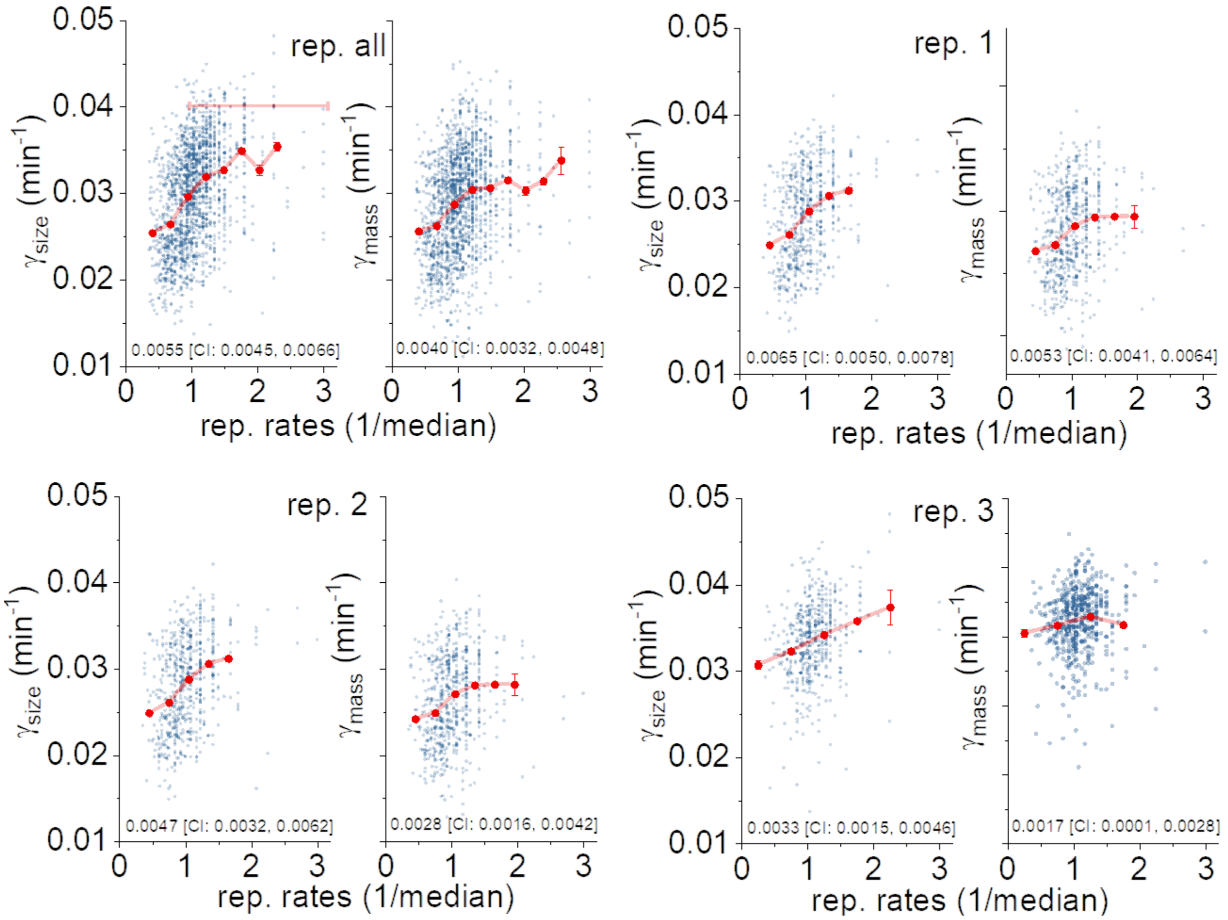

Size (γ_{size}) and mass (γ_{mass}) accumulation rates as a function of the single cell replication rates for all replicates combined and each individual separate; *blue dots* represent the experimental data; *red line* represents the binned data; *legend* displays the slope and the 95% confidence intervals (CI) of the linear fit (by bootstrapping). In the pooled replicate graph (rep. all, *upper left*), the red error bar represents the range in reproduction rates for γ_{size} = 0.041 min^{-1}.

Supplementary Figure 13

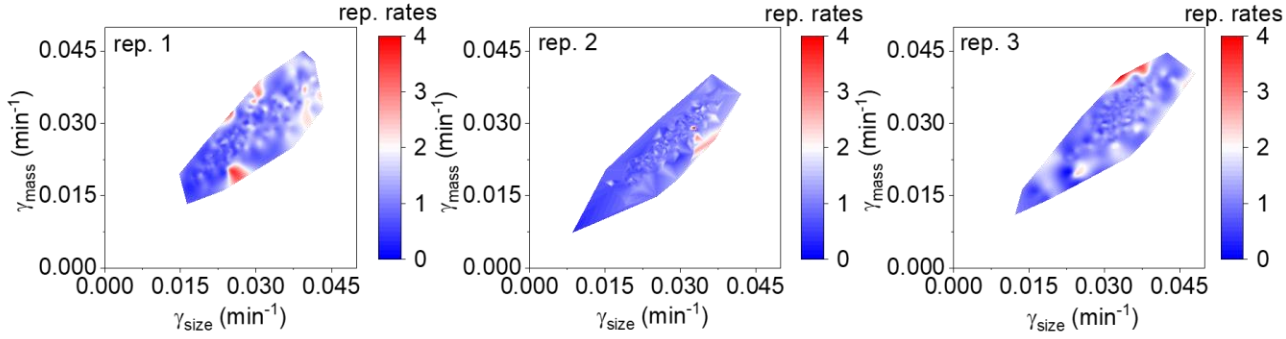

Growth differentiation (i.e., the relationship between γ_{size} and γ_{mass}), with each single-cell observation color-coded by its reproduction rates (normalized over the population median); each graph displays the response of each experimental replicate separately.

Supplementary Figure 14

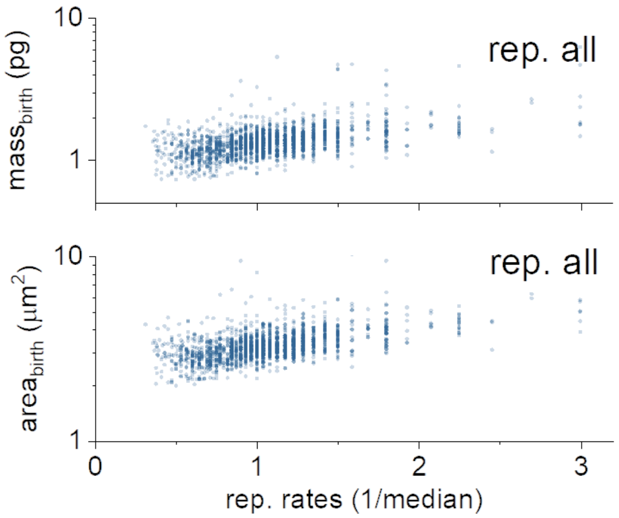

Single cell mass (*upper*) and size (*lower*) at birth as a function of the reproduction rate for the same cell. Note that all triplicates are pooled together and that the y-axis is plotted in logarithmic scale.

Supplementary Figure 15

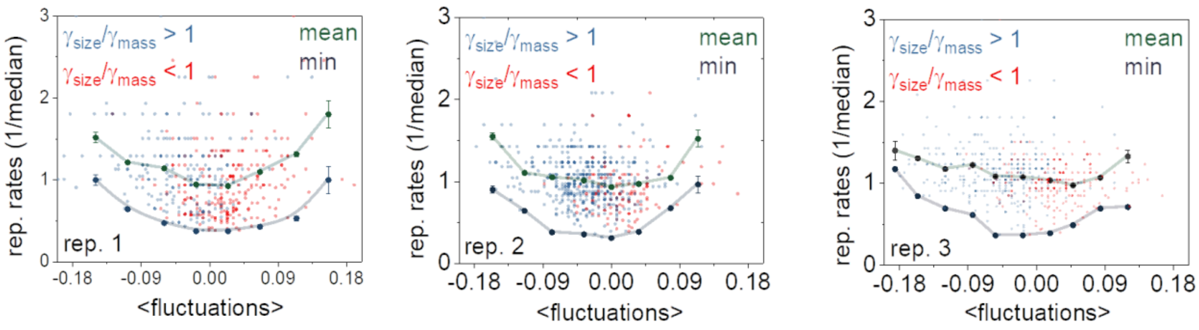

Single cell replication rates (normalized to the population median) plotted as a function of density fluctuations; *blue* and *red* data points represent single cell observations (color coded by their differentiation strategy, $\gamma_{size}/\gamma_{mass}$); *green* and *purple* points represent the averaged binned data and minimum fitness levels at different levels of fluctuations; each graph plots the response of each experimental replicate separately.

Supplementary Figure 16

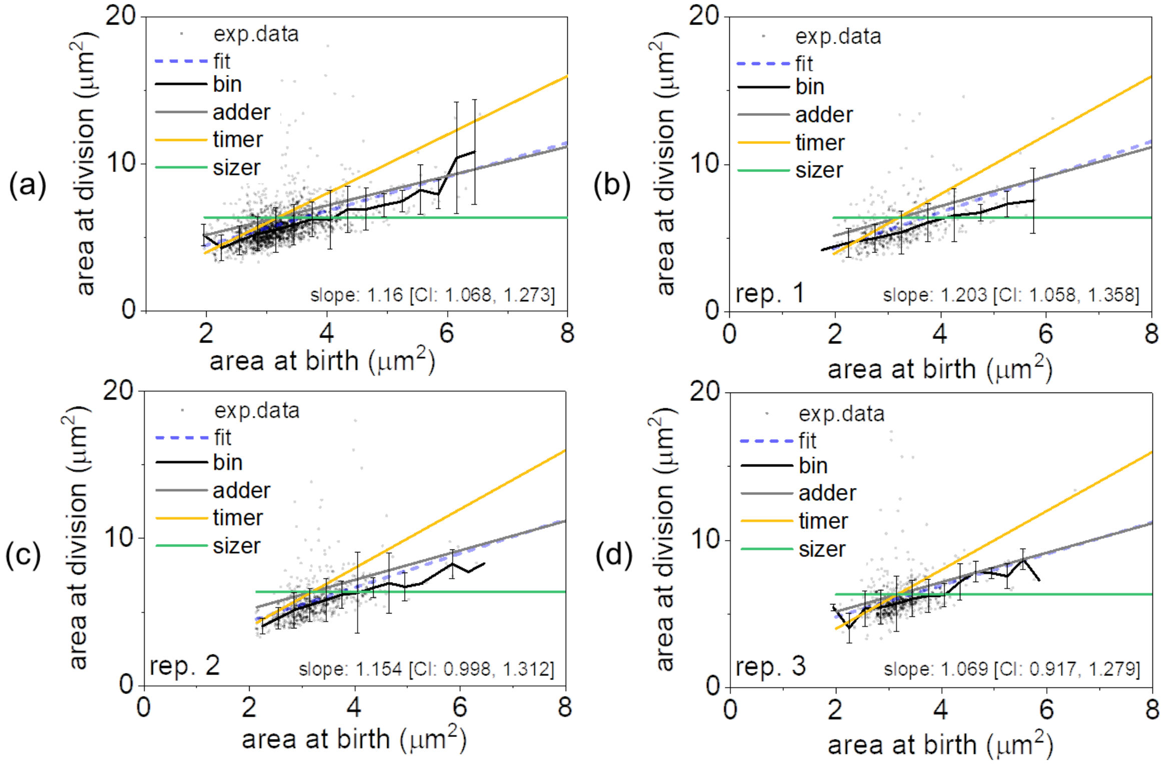

Comparison of the empirical observations of cell size regulation with the adder (*grey*), timer (*yellow*), and sizer (*green*) models. Scatter plots indicate the experimental data; *blue* dotted line represents the linear regression fit (*legend* denotes the slope and the corresponding 95% confidence intervals – CI – determined by bootstrapping); *black* line represents the binning result with the error-bars noting the standard deviation. The adder model appears to best fit to the experiment for both the cumulative response of the combined replicates **(a)** and the individual replicates themselves **(b-d)**.

Supplementary Figure 17

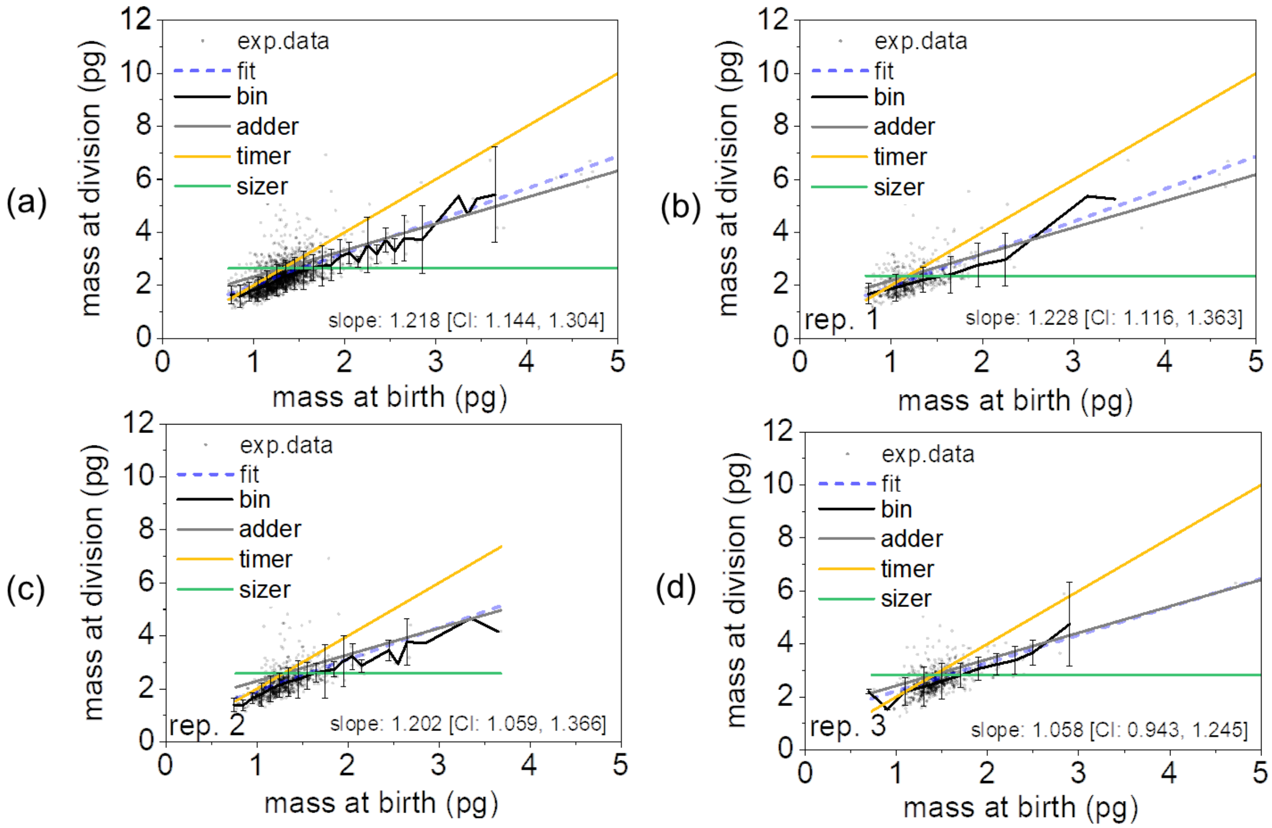

Comparison of the empirical observations of cell mass regulation with the adder (*grey*), timer (*yellow*), and sizer (*green*) models. Scatter plots indicate the experimental data; *blue* dotted line represents the linear regression fit (*legend* denotes the slope and the corresponding 95% confidence intervals – CI – determined by bootstrapping); *black* line represents the binning result with the error-bars noting the standard deviation. The adder model appears to best fit to the experiment for both the cumulative response of the combined replicates **(a)** and the individual replicates themselves **(b-d)**.

Supplementary Figure 18

(a) (b)

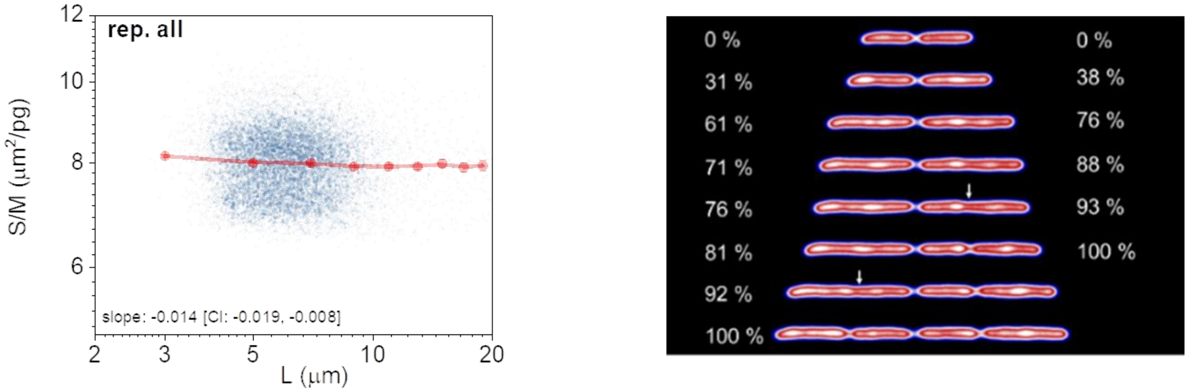

(a) Cell surface area (S) over cell mass (M) as a function of cell length (L); data points represent the experimental data and red line represents the averaged binned data (error bars represent the standard error of the mean). **(b)** Two daughters born at the same time but dividing at different timepoints (100%); white arrows display the constriction formation prior to cell separation.

Supplementary Figure 19

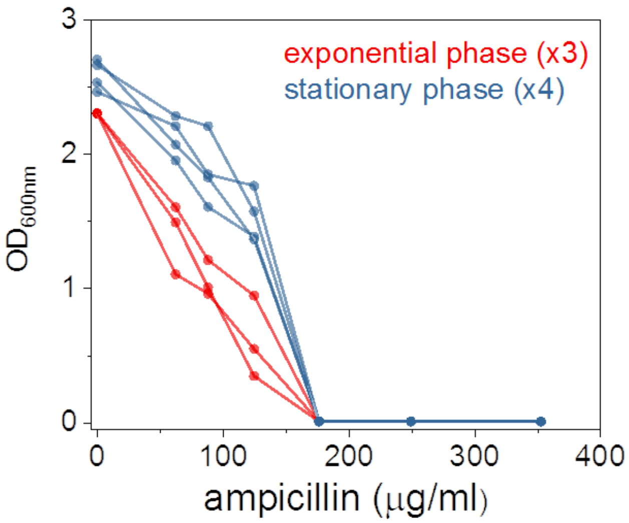

Minimum inhibitory concentration (MIC) determination for the ampicillin resistant E212K mutant using 3 replicates from mid-exponential phase and 4 replicates from stationary phase. No growth was observed at 176 μg/ml ampicillin concentration in all experiments.

Supplementary Figure 20

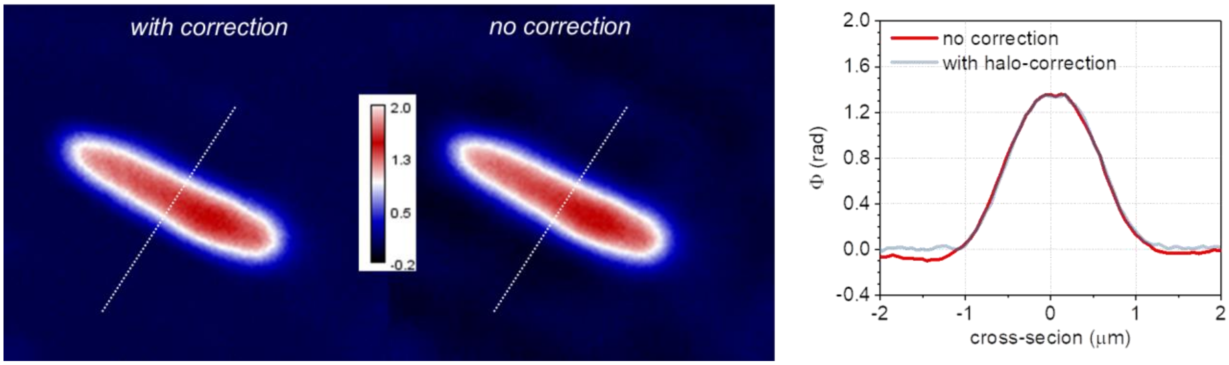

Computational halo correction on the phase profile of a single *E. coli* cell. On the left, the phase images with and without halo correction of the same cell are displayed, while on the right the phase profile along the dotted line is plotted for each image. Halo correction improves the background uniformity and enables better definition of the cell contour, which is critical for cell segmentation.

Supplementary Figure 21

(a)

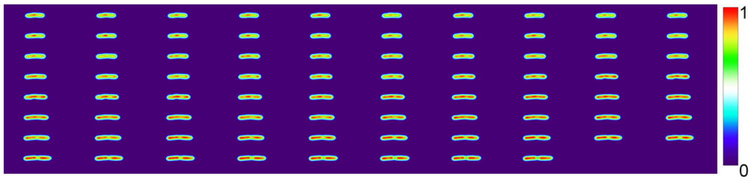

(b)

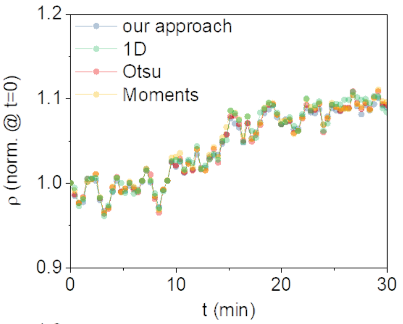

(c)

Pearson's correlations	result
our approach - 1D line	$\rho = 0.993$, $p = 5.82 \times 10^{-71}$
our approach - Otsu	$\rho = 0.996$, $p = 1.69 \times 10^{-80}$
our approach - Moments	$\rho = 0.995$, $p = 2.49 \times 10^{-78}$

(d)

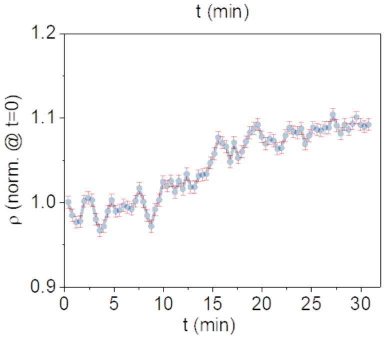

(a) High temporal resolution observations of the density dynamics of a single *E. coli* cell. These measurements were taken every 24 seconds. Other than the sampling frequency, the experiment was performed under identical conditions as the data presented in the manuscript (DH5α strain, 1D immobilization in index matched polymers, nutrient supply from top-integrated gels, constant temperature at 37°C, and 63× magnification objective). **(b)** Comparison of the single-cell density quantified via the method presented in this work with alternative segmentation algorithms, including Otsu and Moments. Our method is also compared against a 1D segmentation approach that is independent of conventional thresholding algorithms and, thus, possible errors in area segmentation. While there were slight differences between different methods in the cellular density as determined by each method, all graphs follow the same trend and greatly overlap when normalized at t = 0, with a greater than 99% Pearson correlation coefficient ($p < 0.001$), as displayed in **(c)**. **(d)** Same observations (with the method applied in all experiments), where the blue line denotes the density of the cell and red lines correspond to the error bars reflecting a potential experimental error in plane selection.

Supplementary Table 1

sample	test	result
replicate 1	Mann-Whitney Test	U = 5657.5; Z = -13.93996; p = 3.62127E-44;
replicate 1	Kolmogorov-Smirnov Test	D = 0.61739; Z = 6.48036; p = 2.85051E-37;
replicate 1	Two sample t Test (Welch Correction)	t = -5.79451; DF = 208; p = 2.51094E-8;
replicate 2	Mann-Whitney Test	U = 21376; Z = -3.31744; p = 9.08477E-4;
replicate 2	Kolmogorov-Smirnov Test	D = 0.17482; Z = 1.6869; p = 0.00576;
replicate 2	Two sample t Test (Welch Correction)	t = -3.5805; DF = 175.03132; p = 4.44038E-4;
replicate 3	Mann-Whitney Test	U = 22010; Z = -4.5231; p = 6.09408E-6;
replicate 3	Kolmogorov-Smirnov Test	D = 0.241; Z = 2.58919; p = 2.25247E-6;
replicate 3	Two sample t Test (Welch Correction)	t = 4.10102; DF = 380.67584; p = 5.03225E-5;
all replicates	Mann-Whitney Test	U = 201838; Z = -7.47378; p = 7.79249E-14;
all replicates	Kolmogorov-Smirnov Test	D = 0.19379; Z = 3.6008; p = 8.70354E-12;
all replicates	Two sample t Test (Welch Correction)	t = 7.6604; DF = 1011.57947; p = 4.32583E-14;

Various statistical significance tests between growth differentiation ($\gamma_{size}/\gamma_{mass}$) and cellular dry-density at birth (ρ_{birth}). All tests denote that differentiation depends on the density at birth with high statistical significance.

Supplementary Table 2

sample	test	result
replicate 1	One Sample Wilcolxon Signed Rank Test	W = 50403; Z = 15.43098; p = 0;
replicate 2	One Sample Wilcolxon Signed Rank Test	W = 50086; Z = 15.40666; p = 0;
replicate 3	One Sample Wilcolxon Signed Rank Test	W = 49455; Z = 15.35789; p = 0;
all replicates	One Sample Wilcolxon Signed Rank Test	W = 554931; Z = 28.10911; p = 0;

One sample Wilcolxon signed rank tests of the non-zero density different between mothers and daughters upon mitosis.
