# Supplementary material for: Density fluctuations, homeostasis, and reproduction effects in bacteria
Source: Commun Biol. 2022 Apr 28;5:397. doi: 10.1038/s42003-022-03348-2 (PMC9050864; doi:10.1038/s42003-022-03348-2)
Supplement: Supplementary file 5 — Reporting Summary [file 42003_2022_3348_MOESM5_ESM.pdf]

nature portfolio

Corresponding author(s): Andreas Vasdekis

Last updated by author(s): Mar 30, 2022

Reporting Summary

Nature Portfolio wishes to improve the reproducibility of the work that we publish. This form provides structure for consistency and transparency in reporting. For further information on Nature Portfolio policies, see our Editorial Policies and the Editorial Policy Checklist.

Statistics

For all statistical analyses, confirm that the following items are present in the figure legend, table legend, main text, or Methods section.

n/a	Confirmed	
☐	☒	The exact sample size (*n*) for each experimental group/condition, given as a discrete number and unit of measurement
☐	☒	A statement on whether measurements were taken from distinct samples or whether the same sample was measured repeatedly
☐	☒	The statistical test(s) used AND whether they are one- or two-sided<br>*Only common tests should be described solely by name; describe more complex techniques in the Methods section.*
☐	☒	A description of all covariates tested
☐	☒	A description of any assumptions or corrections, such as tests of normality and adjustment for multiple comparisons
☐	☒	A full description of the statistical parameters including central tendency (e.g. means) or other basic estimates (e.g. regression coefficient) AND variation (e.g. standard deviation) or associated estimates of uncertainty (e.g. confidence intervals)
☐	☒	For null hypothesis testing, the test statistic (e.g. *F*, *t*, *r*) with confidence intervals, effect sizes, degrees of freedom and *P* value noted<br>*Give P values as exact values whenever suitable.*
☒	☐	For Bayesian analysis, information on the choice of priors and Markov chain Monte Carlo settings
☒	☐	For hierarchical and complex designs, identification of the appropriate level for tests and full reporting of outcomes
☐	☒	Estimates of effect sizes (e.g. Cohen's *d*, Pearson's *r*), indicating how they were calculated

Our web collection on statistics for biologists contains articles on many of the points above.

Software and code

Policy information about availability of computer code

Data collection	Metamorph Version 7.8.13.0, Cell Vista Pro
Data analysis	Origin Pro 2017 64-bit, Matlab R2018a

For manuscripts utilizing custom algorithms or software that are central to the research but not yet described in published literature, software must be made available to editors and reviewers. We strongly encourage code deposition in a community repository (e.g. GitHub). See the Nature Portfolio guidelines for submitting code & software for further information.

Data

Policy information about availability of data

All manuscripts must include a data availability statement. This statement should provide the following information, where applicable:

- Accession codes, unique identifiers, or web links for publicly available datasets
- A description of any restrictions on data availability
- For clinical datasets or third party data, please ensure that the statement adheres to our policy

Key data generated or analyzed during this study are included in this article (and its Supplementary Information), as well as Supplementary Data 1; all data are available from the corresponding author upon reasonable request.

Field-specific reporting

Please select the one below that is the best fit for your research. If you are not sure, read the appropriate sections before making your selection.

☒ Life sciences ☐ Behavioural & social sciences ☐ Ecological, evolutionary & environmental sciences

For a reference copy of the document with all sections, see nature.com/documents/nr-reporting-summary-flat.pdf

Life sciences study design

All studies must disclose on these points even when the disclosure is negative.

Sample size	Sample sizes (namely individual cell dynamic observations per biological replicate) are detailed in figure legends, Methods, and Supplementary Information. Sample size selection was guided by previous quantitative phase imaging and single-cell investigations, as detailed in the Methods section.
Data exclusions	Some data were excluded from the analysis and primarily focus on the ampicillin pressure experiments; in this context, we excluded observations pertaining to single cells undergoing immediate death or growth arrest prior to and during ampicillin pressure, as detailed in the manuscript.
Replication	Each live cell experiment was performed using 3 independent cultures. All three biological replicates displayed very consistent growth dynamics, determined by their growth curves. All statistics reported in the manuscript are based on these 3 biologically independent replicates. MIC experiments were repeated 3x for cells at exponential phase and 4x for cells at stationary phase using independent cultures.
Randomization	Images with single cell resolution were acquired randomly per experimental condition and biological replicate. The analysis of the data resulting from these images was performed randomly as well.
Blinding	Single-cell imaging was performed by automated methods and image analysis was performed by quasi-automated methods. To ensure that data analysis was unbiased, we first processed all images then analyzed the resulting data.

Reporting for specific materials, systems and methods

We require information from authors about some types of materials, experimental systems and methods used in many studies. Here, indicate whether each material, system or method listed is relevant to your study. If you are not sure if a list item applies to your research, read the appropriate section before selecting a response.

Materials & experimental systems

n/a	Involved in the study
☒	☐ Antibodies
☒	☐ Eukaryotic cell lines
☒	☐ Palaeontology and archaeology
☒	☐ Animals and other organisms
☒	☐ Human research participants
☒	☐ Clinical data
☒	☐ Dual use research of concern

Methods

n/a	Involved in the study
☒	☐ ChIP-seq
☒	☐ Flow cytometry
☒	☐ MRI-based neuroimaging
